# Alzheimer’s disease risk factors as mediators of subjective memory impairment and objective memory decline: protocol for a construct-level replication analysis

**DOI:** 10.1186/s12877-018-0954-5

**Published:** 2018-10-29

**Authors:** Nikki L. Hill, Jacqueline Mogle

**Affiliations:** College of Nursing, The Pennsylvania State University, 201 Nursing Sciences Bldg, University Park, PA 16802 USA

**Keywords:** Cognition, Memory, Self-report, Subjective memory, Alzheimer’s disease

## Abstract

**Background:**

Subjective memory impairment (SMI), or the perception of memory problems in the absence of objective memory deficits, is associated with negative outcomes of individual and societal significance, including a substantially increased risk of Alzheimer’s disease (AD). However, little is known regarding the mediators that link SMI and memory decline in some individuals, or which older adults with SMI are at greatest risk for memory decline. In this study, we will examine modifiable AD risk factors (specifically affective symptoms and activity participation) as mediators underlying linkages among SMI and memory decline over time; furthermore, we will characterize SMI subgroups at highest risk for memory decline via this pathway.

**Methods:**

This study utilizes a series of construct-level replication analyses across four large longitudinal datasets to maximize the unique aspects of each dataset as well as test the reproducibility of findings across multiple populations to establish generalizability. The current study’s sample (*n* > 40,000) is drawn from the Einstein Aging Study, Health and Retirement Study, Minority Aging Research Study, and National Health and Aging Trends Study. Participants must meet the following basic criteria for inclusion: age 55 or older and no evidence of cognitive impairment at baseline. We will use multilevel modeling to determine whether higher levels of SMI are related to increased affective symptoms and decreased activity participation, as well as whether this relationship is moderated by neuroticism, family history of AD, and race/ethnicity. Finally, we will test our full conceptual model that examines whether changes in affective symptoms and activity participation mediate the relationship between SMI and objective memory decline. Specifically, we will test moderated mediation as we hypothesize these relationships to hold among subgroups of older adults.

**Discussion:**

Discovery of modifiable AD risk factors that mediate the association between SMI and memory decline (the earliest and most central deficit in AD) will provide explicit, and potentially novel, targets for intervention. Additionally, identifying individuals at highest risk for negative reactions to SMI will serve to enrich samples for future research as well as to help guide the development of SMI assessment tools to identify older adults at greatest risk for debilitating outcomes.

**Electronic supplementary material:**

The online version of this article (10.1186/s12877-018-0954-5) contains supplementary material, which is available to authorized users.

## Background

Alzheimer’s disease (AD) is, by definition, a progressive disease with an insidious onset. In its preclinical phase, pathological changes accumulate for a decade or more prior to clinical presentation of cognitive deficits [1]. Subjective memory impairment (SMI), the report of memory impairment with no clinical indication of memory deficit [2], often precedes clinically identifiable memory deficit early in the course of AD [3]. Affected individuals become aware of functional changes that may be too subtle for clinical testing to detect, or are most obvious in complex, real-world environments [4–6]. Over 20% of community-dwelling older adults experience SMI [7, 8], and this group is up to four times as likely to develop mild cognitive impairment or AD within the next 7 years [9]. However, given that SMI does not always lead to AD, there is much we do not yet understand regarding SMI as a preclinical indicator of actual memory decline. Little is known regarding which individuals with SMI are at highest risk for developing AD or what individual factors influence the trajectory of cognitive decline [10].

Beyond an increased AD risk, SMI is associated with decreased participation in cognitive, physical, and social activities [11–14] as well as higher levels of affective (depressive or anxiety) symptoms [15]. Known AD risk factors include non-modifiable factors such as low educational attainment and genetic predisposition, but also modifiable factors such as depression, physical inactivity, lack of social connectedness, and a less cognitively complex lifestyle [16–18]. Affective symptoms and lower physical, cognitive, and social activity participation are known to co-occur with SMI and may result from negative reactions to the experience of SMI. For example, concerns about memory decline may precipitate depressive symptoms [19, 20] or withdrawal from cognitively protective behaviors such as participating in social activities [21]. Understanding these relationships is of critical importance since affective symptoms and lower activity participation are implicated in increased AD risk. Furthermore, older adults’ reactions to SMI may influence mental health (i.e., psychological mechanisms such as affective symptoms) and lifestyle (i.e., behavioral mechanisms such as decreases in activity participation). Depending on whether these reactions are positive (e.g., proactively increasing participation in cognitively complex activities leading to neuroprotective and compensatory effects; [22]), or negative (e.g., increased memory-related anxiety leading to the deleterious cognitive effects of chronic stress; [23]) the timeline of clinically identifiable memory deficit along the AD trajectory may be altered.

Evidence suggests that reactions to SMI in older adults are influenced by several individual factors. First, the personality trait of neuroticism (i.e., a persistent tendency to experience negative emotions) is associated with SMI in community [24, 25] and memory clinic [26] samples. Complicating our understanding of neuroticism’s role in the experience of SMI is the increased risk for individuals higher in neuroticism to experience affective symptoms (known risk factors for AD). Longitudinal studies suggest that individuals higher in neuroticism may be more attuned to notice memory problems or less likely to habituate to memory changes [27], and that neuroticism may moderate associations between SMI and psychological as well as physical health [28]. Second, a family history of AD or other dementias is associated with increased worry about SMI [29]. Fear of developing AD is common among older adults, and its impact is influenced by personal proximity to the disease: a family history of AD and/or experience as an informal caregiver to a family member with AD increases older adults’ AD-related fears [30]. Third, lower levels of AD knowledge among Blacks compared to Whites [31] as well as AD diagnoses occurring at more advanced disease stages in Blacks [32], suggest that perceptions of SMI may differ among racial or ethnic groups. Therefore, individual characteristics are likely important to consider when examining relationships among SMI, modifiable AD risk factors, and memory decline over time.

Complicating the interpretation of existing evidence regarding SMI is the heterogeneity of assessment approaches across studies. These range from single-item measures to extensive symptom checklists, self-ratings of memory abilities to endorsements of memory concerns, and comparisons to one’s self over time to comparisons with others [33]. In their review of subjective cognitive decline measures employed in preclinical AD investigations, Rabin and colleagues urge caution in comparing findings across studies due to the substantial heterogeneity of measures [34]. There is an identified need to interpret findings that associate SMI and adverse outcomes with specificity: item construction matters.

The experience of SMI is associated with negative outcomes of great individual and societal significance, including an increased risk of AD. Current evidence also supports relationships between SMI and the AD risk factors of increased depressive and anxiety symptoms as well as decreased cognitive, physical, and social activity. However, no research to date has: 1) elucidated mediators underlying linkages among SMI and memory decline; 2) characterized SMI subgroups at highest risk by identifying moderators of these mediational relationships; or 3) compared the differential predictive effects of specific SMI assessment items. This study addresses each of these critical knowledge gaps through the testing of our conceptual model using multilevel modeling in construct-level replication analyses [35, 36] across four longitudinal datasets.

### Conceptual framework

**Fig. 1:**
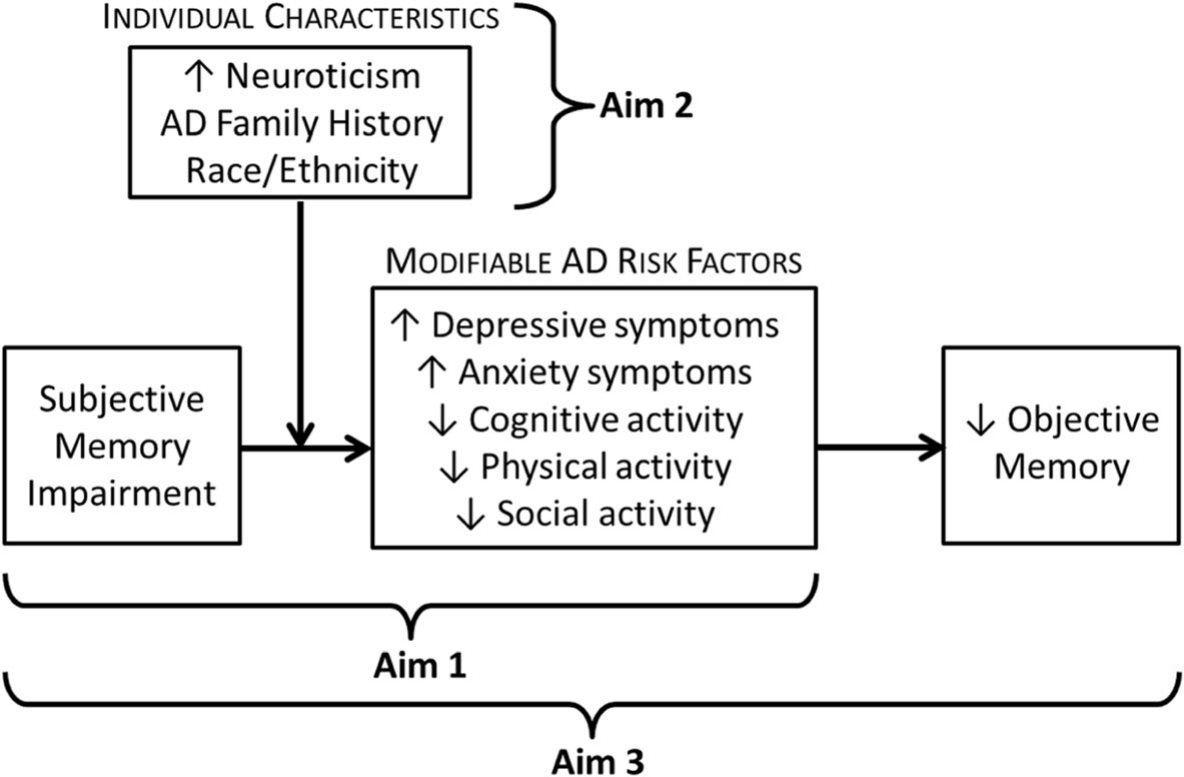
Conceptual model guiding aims

SMI precipitates worry about AD [37, 38], depressive symptoms [39, 40], and withdrawal from cognitively protective activities (e.g., physical or social activities; [11]). These negative reactions to SMI represent one potential pathway by which SMI can contribute to AD development since both affective symptoms [41, 42] and reduced activity participation [43–46] are associated with increased AD risk. Furthermore, these potential reactions are also considered modifiable AD risk factors and opportunities for intervention to delay functional decline in older adults. The conceptual model guiding this study (Fig. 1) posits that, for some older adults, changes in modifiable AD risk factors mediate the relationship between SMI and objective memory decline. This model was developed based on three bodies of evidence: 1) SMI is associated with several modifiable AD risk factors, specifically increases in affective symptoms and reduced activity participation; 2) SMI is associated with an increased risk of objective memory decline; and, 3) the modifiable AD risk factors of interest in this study are associated with objective memory decline. Our conceptual model theorizes that negative reactions to SMI by high-risk older adults lead to an increase in modifiable AD risk factors (i.e., increased affective symptoms and reduced activity participation), subsequently leading to objective memory decline over time.

Additionally, our model considers individual differences that influence reactions to SMI, which is particularly important for identifying older adults at highest risk and ultimately tailoring intervention strategies. Higher neuroticism is consistently associated with SMI [4] and affective symptoms [47], and episodes of forgetfulness induce emotional distress and worry about developing AD in some older adults [48], particularly among those with a family history of AD [29, 30]. SMI predicts AD risk [49] and memory decline [50] for subgroups of older adults. Our conceptual model proposes that individual characteristics (e.g., personality and family history of AD) influence older adults’ reactions to SMI, and therefore moderate the relationship between SMI and modifiable AD risk factors. Due to the complex interplay of SMI and a chain of risk factors leading to AD, establishing moderators of these relationships will help us to determine susceptibility to negative reactions [51] and support interventions that focus on individual needs and risk profiles.

### Study aims

The overall goals of this study are to characterize subgroups of older adults with SMI for whom these AD risk factors are more likely to develop over time, and to explicate longitudinal relationships among SMI, modifiable AD risk factors, and objective memory decline for subgroups of older adults. We will use construct-level replication analyses across multiple longitudinal datasets to investigate the following study aims:

- Aim 1: Test the longitudinal predictive utility of SMI on modifiable AD risk factors.
- Aim 2: Identify moderators of the relationships between SMI and modifiable AD risk factors.
- Aim 3: Test a moderated mediation model where individual characteristics (e.g., neuroticism) moderate the relationship between SMI and modifiable AD risk factors (e.g., depressive symptoms) that in turn mediate objective memory decline.

## Methods

This study applies multilevel modeling (MLM) in construct-level replication analyses [35, 36] across four datasets collected in ongoing longitudinal studies of aging: Einstein Aging Study (EAS), Health and Retirement Study (HRS), Minority Aging Research Study (MARS), and National Health and Aging Trends Study (NHATS). These datasets represent a diverse group of over 40,000 adults (ages 55 and older) across follow-up periods of up to 22 years. As data collection intensive projects that obtain a wide variety of individual measures, data from longitudinal studies can be used to address questions about within-person processes as well as covariation, change, and between-person variability among these processes over time [36]. When carried out in large, diverse samples such as in the datasets in this study, we can examine developmental processes while also allowing for individual differences in these processes, rather than assuming equivalence in trajectories across all individuals. An additional advantage of this approach is to test the reproducibility of findings across multiple samples to establish the generalizability of the developed conceptual model.

### Sample characteristics

Participants must meet the following basic criteria for inclusion in the study: age 55 or older, no clinical diagnosis of mild cognitive impairment or dementia at baseline (when available), and no objective memory impairment at baseline (when a diagnosis is not available). Brief descriptions of the samples and sampling strategy for each of the four studies are presented in Additional file 1: Table S1, including the approximate number of participants (total *n* = 47,253) meeting the current study’s basic inclusion criteria. These studies used a variety of sampling methodologies, including two large samples representative of the U.S. population of older adults (HRS and NHATS). MARS recruits exclusively African Americans, and EAS captures extensive follow up with the oldest old (85+ years) who are at greater risk for memory decline.

### Designs and procedures of selected studies

The datasets for this project were collected within studies that are each uniquely relevant to achieving the study aims. EAS is a longitudinal cohort study examining cognitive aging, AD, and other dementing disorders among community-dwelling older adults in an urban, multi-ethnic area of New York City [52]. Data collection began in 1993 and occurs annually via in-person comprehensive medical and neuropsychological examinations. HRS is a nationally representative longitudinal survey of individuals age 50 or older and provides a wealth of data on health, cognition, work and retirement, and psychosocial information [53]. Data collection began in 1992 and occurs biennially; samples are replenished every 6 years. As noted in Additional file 1: Table S1, the HRS sample is a multi-stage probability design that includes geographic stratification and oversampling of certain demographic groups. The survey is conducted as a mixed-mode design in both in-person and telephone formats, depending on timing (baseline or follow-up year) and age of participants. Since HRS includes participants aged 50 or older, we will limit our HRS dataset to ages 55 and older only. MARS is a longitudinal cohort study examining cognitive decline and AD in older African Americans exclusively in suburban and urban areas of Chicago, IL [54]. Data collection began in 2004 and occurs annually via in-person comprehensive clinical and neuropsychological examinations. Community engagement and relationship-building has been a key component of MARS recruitment, leading to a unique sample representative of African Americans with diverse demographic profiles. Similar to HRS, NHATS is meant to provide a national resource for investigating aging with the collection of data on physical and cognitive capacity, activities of daily life, and participation in valued activities [55]. Data collection began in 2011 and occurs annually via in-person interviews in a nationally representative sample of Medicare beneficiaries, with oversamples of older and Black individuals.

### Measures

The selected datasets include one or more assessments of SMI, AD risk factors (affective symptoms and activity participation), individual characteristics of interest (personality, family history of AD, race/ethnicity), and objective measures of memory performance. Additional file 2: Table S2 compares the instruments or measurement items available in each dataset for the concepts of interest in the current study. The heterogeneity of SMI measures across studies is a known limitation of existing research, and the selected datasets for this study are no exception. SMI measures utilized in this construct-level replication analysis include self-ratings of current memory performance (all four studies), comparisons to one’s past memory performance over varying time periods (all four studies), comparison to others of the same age (one study), and rating of the interference of memory problems with important or daily activities (two studies).

### Analysis plan

We will use MLM to determine whether higher levels of SMI are related to increased affective symptoms and decreased activity participation (Aim 1) and whether this relationship is moderated by neuroticism, family history of AD, and race/ethnicity (Aim 2). Finally, we will test our full conceptual model that examines whether changes in affective symptoms and activity participation mediate the relationship between SMI and objective memory decline (Aim 3). Specifically, we will test moderated mediation as we hypothesize these relationships to hold among subgroups of older adults which will be identified in Aim 2.

#### General approach to analysis

Construct-level replication is a coordinated analytic approach that assumes consistency of construct representation across the datasets, rather than consistency of construct measurement required by stricter analytic techniques (e.g., mega-analysis). The selected datasets include measures that broadly reflect the constructs of interest for the current analyses though measured through different, standardized methods. Measures will be harmonized to the extent possible (e.g., similar scoring for items that are consistent across datasets) to allow for comparison of results and conclusions across datasets. However, a benefit to construct-level replication is the ability to probe differences that are primarily due to measurement; this is critical due to the differences across the datasets in the SMI measures used.

SMI items that rely on a person’s perception of current memory ability tend to have stronger links to functional activities while SMI items that depend on an individual’s memory-related concerns tend to have stronger links to affective symptoms [56, 57]. We hypothesize that items assessing current memory problems or current interference of memory problems with important activities reflect memory-related abilities (e.g., frequency of current memory problems), and that these items will be more strongly associated with changes in activity participation. In contrast, we hypothesize that items assessing memory change over time and items that ask for comparing memory performance to age-appropriate peers reflect ratings of memory concern (i.e., worry that memory has changed or is worse than peers) will be more strongly associated with changes in affective symptoms. We will specifically compare these different types of relationships within and across datasets as part of the analytic process.

### Other analytic considerations

Similar data preparation techniques will be used across all datasets to facilitate comparisons consistent with the construct-level replication approach. For example, all datasets will use grand mean centering of continuous variables and consistent reference groups (e.g., males when sex is considered as a covariate) for categorical variables. We will use standardized coding for time (i.e., years) to equate the time metric across all datasets as well as calculate standardized estimates to compare the magnitude of effects across datasets. The longitudinal nature of the selected datasets will lead to some attrition over time. All participants will be included in analyses regardless of amount of follow up. Pattern mixture modeling will be used in sensitivity analyses to determine whether patterns of missingness impact substantive conclusions.

#### Analytic approach for aim 1: Test the longitudinal predictive utility of SMI on modifiable AD risk factors

Using MLM we will first determine whether SMI, affective symptoms, and activity participation show significant within-person covariation over time. This analysis will determine whether at assessment occasions when an individual reports greater (compared to lesser) SMI severity, they also report greater affective symptoms and reduced activity participation. Next, we will test autoregressive MLMs. In these models, SMI from a previous occasion will be used to predict future modifiable AD risk factors of interest. We will also test whether these relationships are reciprocal by examining how affective symptoms (or activity participation) predict future reports of SMI. In these models, concurrent and lagged affective symptoms/activity participation (centered at baseline) will be used to predict SMI. These analyses will allow us to determine whether the relationships among SMI and the modifiable AD risk factors of interest are unidirectional.

#### Analytic approach for aim 2: Identify moderators of the relationships between SMI and modifiable AD risk factors

Extending the analyses described for Aim 1, we will add interactions with neuroticism, family history of AD, and race/ethnicity in separate MLMs to determine whether these individual characteristics increase the severity of negative responses to SMI (i.e., the modifiable AD risk factors of interest: affective symptoms and activity participation). The moderators selected are between-person variables and will be entered as level 2 predictors in all equations tested.

#### Analytic approach for aim 3: Test a moderated mediation model where individual characteristics (e.g., neuroticism) moderate the relationship between SMI and modifiable AD risk factors (e.g., depressive symptoms) that in turn mediate objective memory decline

**Fig. 2:**
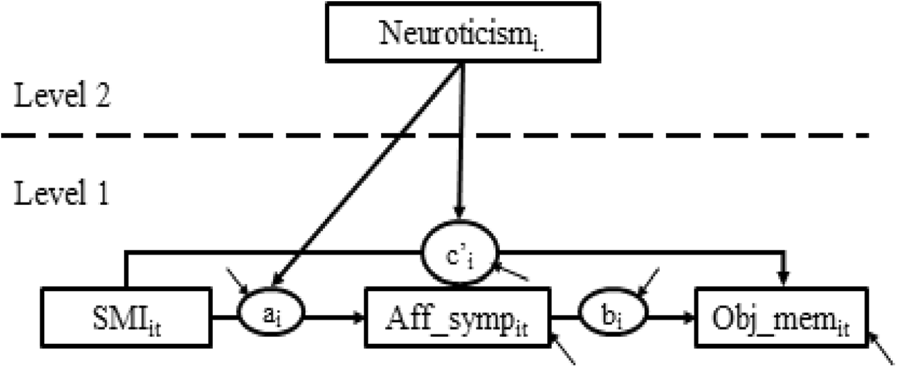
Moderated multilevel mediation

Finally, we will test moderated multilevel mediation [58]. As an example (Fig. 2), we examine the extent to which affective symptoms at time *t* for individual *i* account for the relationship between an individual’s level of SMI at time *t* and their objective memory decline over time. SMI and affective symptoms will be baseline-centered to track changes across time within an individual [59]. An individual’s level of neuroticism is included as a moderator in the proposed moderated mediation in Fig. 2. We will test the significance of the c’ path using methods suggested by Selig and Preacher [60]. This method will allow us to test the unique contributions of SMI and affective symptoms (or activity participation) to the prediction of objective memory performance.

## Discussion

The earliest and most central deficit of AD is memory decline [61, 62]. While some aspects of memory, such as procedural memory, are relatively preserved until later disease stages, episodic memory deficits are among the earliest signs of AD and are clinically relevant due to their functional implications [63]. Individuals with SMI are a heterogeneous group. Overall, they are at increased risk for memory decline [50] and AD [2], but most older adults with SMI will not go on to develop AD. Our understanding of the SMI-AD pathway is limited by a paucity of research on the temporal linkages among SMI, reactions to SMI associated with AD risk, and the earliest clinical indicator of AD: objective memory decline. Additionally, we lack knowledge regarding which individual characteristics (e.g., personality, family history of AD) moderate the reactions to SMI that, in turn, mediate memory decline. This study employs an efficient, coordinated analytic approach to address these knowledge gaps and determine: 1) how SMI is associated with several modifiable AD risk factors (affective symptoms and activity participation) as well as declines in memory over time, and 2) how characteristics of individuals with SMI (i.e., personality, family history of AD, and race/ethnicity) influence the strength of the relationships among SMI and the identified modifiable AD risk factors. In addition to explicating the complex relationships at play, identifying moderators of these relationships will allow characterization of highest risk SMI subgroups. This holds important implications for targeting future interventions, enriching samples for future research, and developing effective screening tools.

SMI is the focus of much current research, but these bodies of literature have remained disparate, linking SMI to AD or associating SMI with a variety of poor psychological or behavioral outcomes. There are few longitudinal examinations of these complex relationships in representative community-based samples. Replicating analyses across datasets will provide insight into the durability of the behavioral and psychological mechanisms (i.e., negative reactions to SMI) that increase AD risk among diverse groups of older adults with SMI. Furthermore, applying sophisticated analytic approaches and utilizing existing longitudinal datasets maximizes economy and efficiency.

At the population level, we are challenged with identifying risk profiles, screening tools, and intervention approaches that are applicable to a wide range of clinical settings and can be efficiently implemented in epidemiological research. Focusing on mediators of the relationship between SMI and memory decline over time will address scientific, as well as practical, needs in improving our understanding of preclinical AD. The potential mediators identified in this proposal are modifiable risk factors for AD. Therefore, once we identify these as mediators of the pathway linking SMI to objective memory decline, interventions to prevent decline related to these risk factors can be developed, refined, and tested with specificity and clinical relevance in mind, ultimately combining this knowledge with developing evidence regarding the pathophysiology of AD.

## Additional files

Additional file 1: **Table S1.** Sample Descriptions. (DOCX 18 kb)

Additional file 2: **Table S2.** Study Measures. (DOCX 20 kb)

## Abbreviations

AD: Alzheimer’s disease
EAS: Einstein Aging Study
HRS: Health and Retirement Study
MARS: Minority Aging Research Study
MLM: Multilevel Modeling
NHATS: National Health and Aging Trends Study
SMI: Subjective Memory Impairment
