# Supplementary material for: Alzheimer’s disease risk factors as mediators of subjective memory impairment and objective memory decline: protocol for a construct-level replication analysis
Source: BMC Geriatr. 2018 Oct 29;18:260. doi: 10.1186/s12877-018-0954-5 (PMC6206637; doi:10.1186/s12877-018-0954-5)
Supplement: Supplementary file 1 — Table S1. Sample Descriptions. (DOCX 18 kb) [file 12877_2018_954_MOESM1_ESM.docx]

| **Study** | **Sample Description** | | |  | **Gender** | |  | **Racial Group Representation** | | | **# Participants Meeting Basic Criteria** | **Maximum # Follow-up Waves** |
| --- | --- | --- | --- | --- | --- | --- | --- | --- | --- | --- | --- | --- |
|  | **Sampling Method** | **Population** | **Age** |  | **Female** | **Male** |  | **White** | **Black** | **Other** |  |  |
| EAS | Systematic, probability | Community-dwelling (Bronx, NY) | 70+ |  | 61% | 39% |  | 69% | 31% | 0% | 1962 | 16 (annual) |
| HRS | Multi-stage, probability | Adults 50+ years (U.S.) | 55+ |  | 56% | 44% |  | 79% | 18% | 3% | 36374 | 12 (annual) |
| MARS | Convenience, non-probability | Community-dwelling (Chicago, IL) | 65+ |  | 76% | 24% |  | 0% | 100% | 0% | 672 | 11 (annual) |
| NHATS | Age-stratified, probability | Medicare users (U.S.) | 65+ |  | 52% | 48% |  | 83% | 17% | 0% | 8245 | 6 (annual) |

Additional file 1 Table S1

Table S1 *Sample Descriptions*
