# Supplementary material for: Alzheimer’s disease risk factors as mediators of subjective memory impairment and objective memory decline: protocol for a construct-level replication analysis
Source: BMC Geriatr. 2018 Oct 29;18:260. doi: 10.1186/s12877-018-0954-5 (PMC6206637; doi:10.1186/s12877-018-0954-5)
Supplement: Supplementary file 2 — Table S2. Study Measures. (DOCX 20 kb) [file 12877_2018_954_MOESM2_ESM.docx]

Additional file 2 Table S2

Table S2 Study Measures

|  | **EAS** | **HRS** | **MARS** | **NHATS** |
| --- | --- | --- | --- | --- |
| **Predictors** | | | | |
| **SMI** | - In the past year, how often did you have trouble remembering things – frequently, sometimes, rarely, or never? - Compared with [one year ago/10 years ago], do you have trouble remembering things more often, less often, or about the same? - Comparing important and unimportant things, what % of the time do you forget things that are important to you? - Do you feel that you have more problems with memory than most? | - How would you rate your memory at the present time? Would you say it’s excellent, very good, good, fair, or poor? - Compared to [last survey/two years ago], would you say your memory is better now, about the same, or worse now than it was then? | - About how often do you have trouble remembering things – frequently, sometimes, rarely, or never? - Compared to 10 years ago, would you say that your memory is much worse, a little worse, the same, a little better or much better? | - How would you rate your memory at the present time? Would you say it’s excellent, very good, good, fair, or poor? - In the last month, how often did memory problems interfere with your daily activities? Would you say every day, most days, some days, rarely, or never? - Compared to 1 year ago would you say your memory is much better now, better now, about the same, worse now, or much worse now? |
| **Moderators** | | | | |
| **Personality** | NEO Personality Scales (IPIP) | The "Big 5" Personality Traits (modified MIDUS) | Neuroticism (12-item version from NEO-Five-Factor Inventory) | Personality Traits (adapted from MIDI Personality Scales) |
| **Family History of AD** | Family information | Parents’ memory disease | Clinical Interview | Not Available |
| **Mediators** | | | | |
| **Depressive Symptoms** | GDS | CES-D | CES-D | PHQ-2 |
| **Anxiety Symptoms** | Not Available | Beck Anxiety Inventory | Not Available | GAD-2 |
| **Activity Participation** | Social Networks  Social Support  Social activities  Leisure activities  Physical activities  Cognitive activities | Physical Activities Social Networks Social Activities Cognitive Activities | Physical Activity Late Life Social Activity  Social Networks Late Life Cognitive Activity | Household Activities Medical Care Activities Social Networks Participation in Valued Activities |
| **Outcomes** | | | | |
| **Objective Memory Performance** | Free & Cued Selective Recall Test  WAIS Tests  Verbal and Category Fluency Tests  WRAT Scores Logical Memory Tests from WMS-R  Stroop Tests  Trials Times and Tests | Immediate & Delayed Word Recall  Serial 7’s  Backwards Counting  Date (Month, Day, Year, day of Week)  Name of Current President and Vice President of the U.S.  Object Naming  Vocabulary Scores (Word definition) | Category Fluency Tests  Digits: Backward and Forward Counting and Ordering  Logical Memory Ia & IIa Immediate & Delayed Story Recall Word List Memory Recall & Recognition  Stroop Tests | Date (Month, Day, Year, day of Week)  Immediate & Delayed Word Recall  Name of Current President and Vice President of the U.S.  Clock Drawing Test |

CES-D = Center for Epidemiologic Studies Depression Scale; GAD = Generalized Anxiety Disorder Scale; GDS = Geriatric Depression Scale; IPIP = International Personality Item Pool; MIDUS = Midlife in the United States; SF-36 = 36-item Short Form Health Survey; MIDI = Midlife Development Inventory; PHQ = Patient Health Questionnaire; WMS-R = Wechsler Memory Scale -Revised; WAIS = Wechsler Adult Intelligence Scale; WRAT = Wide Range Achievement Test
